# Supplementary material for: β-cell deletion of the PKm1 and PKm2 isoforms of pyruvate kinase in mice reveals their essential role as nutrient sensors for the KATP channel
Source: eLife. 2022 Aug 23;11:e79422. doi: 10.7554/eLife.79422 (PMC9444242; doi:10.7554/eLife.79422)
Supplement: Figure 1—source data 1. [file elife-79422-fig1-data1.zip › Figure 1-source data 1 western/Western blots.pptx]

## Slide 1
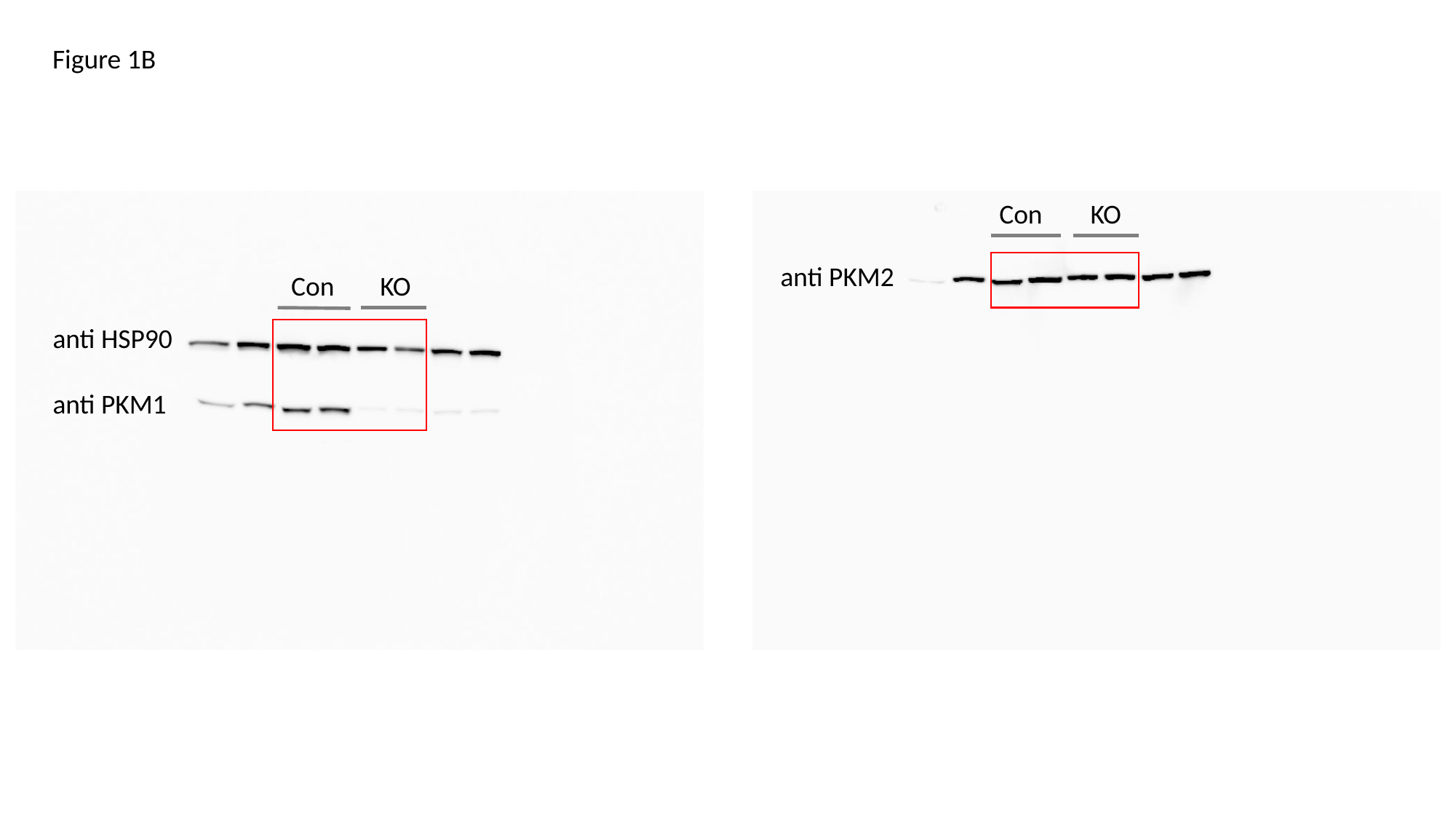

Figure 1B
Con
KO
anti PKM2
Con
KO
anti HSP90
anti PKM1

## Slide 2
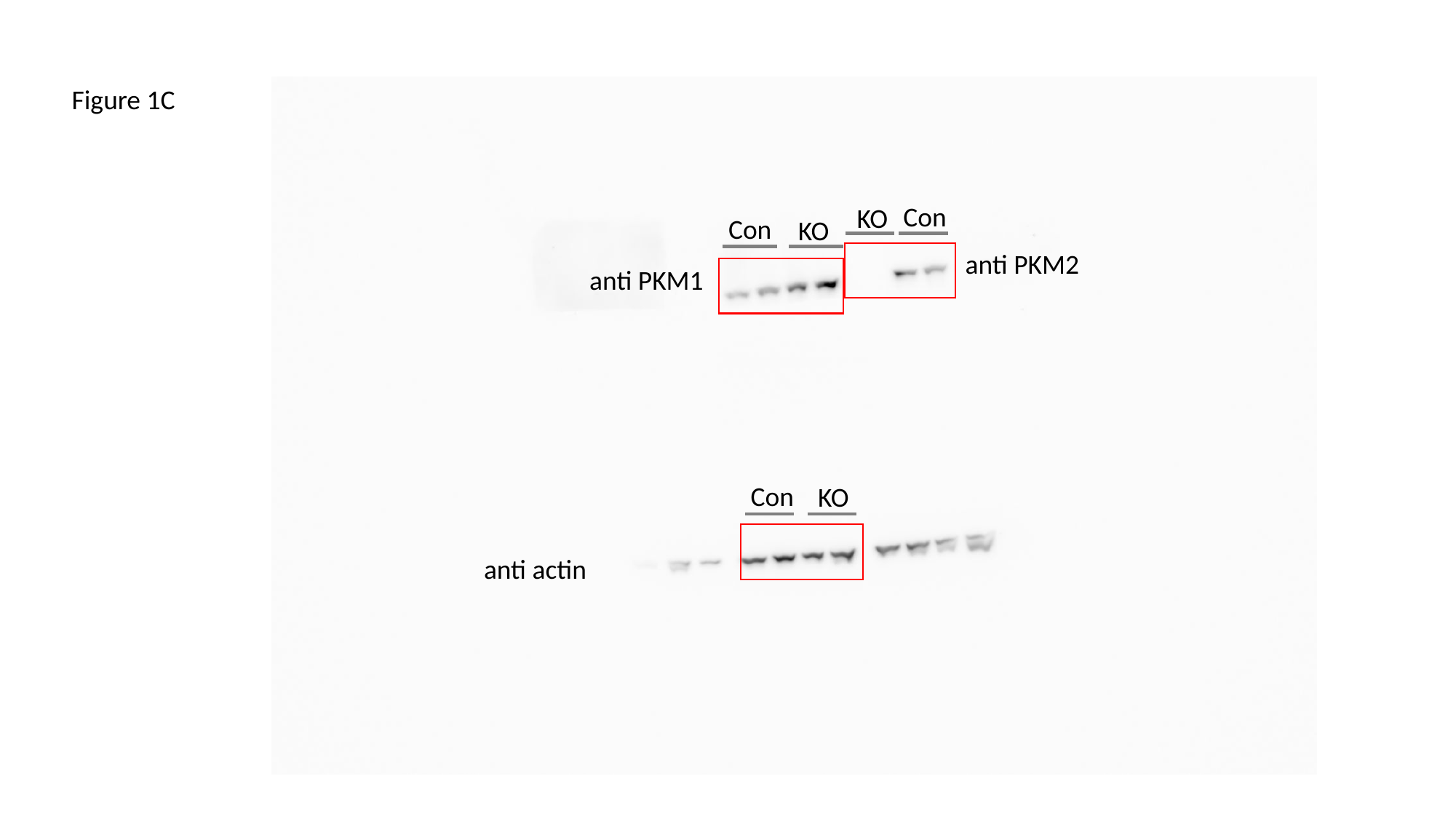

Figure 1C
Con
KO
Con
KO
anti PKM2
anti PKM1
Con
KO
anti actin

## Slide 3
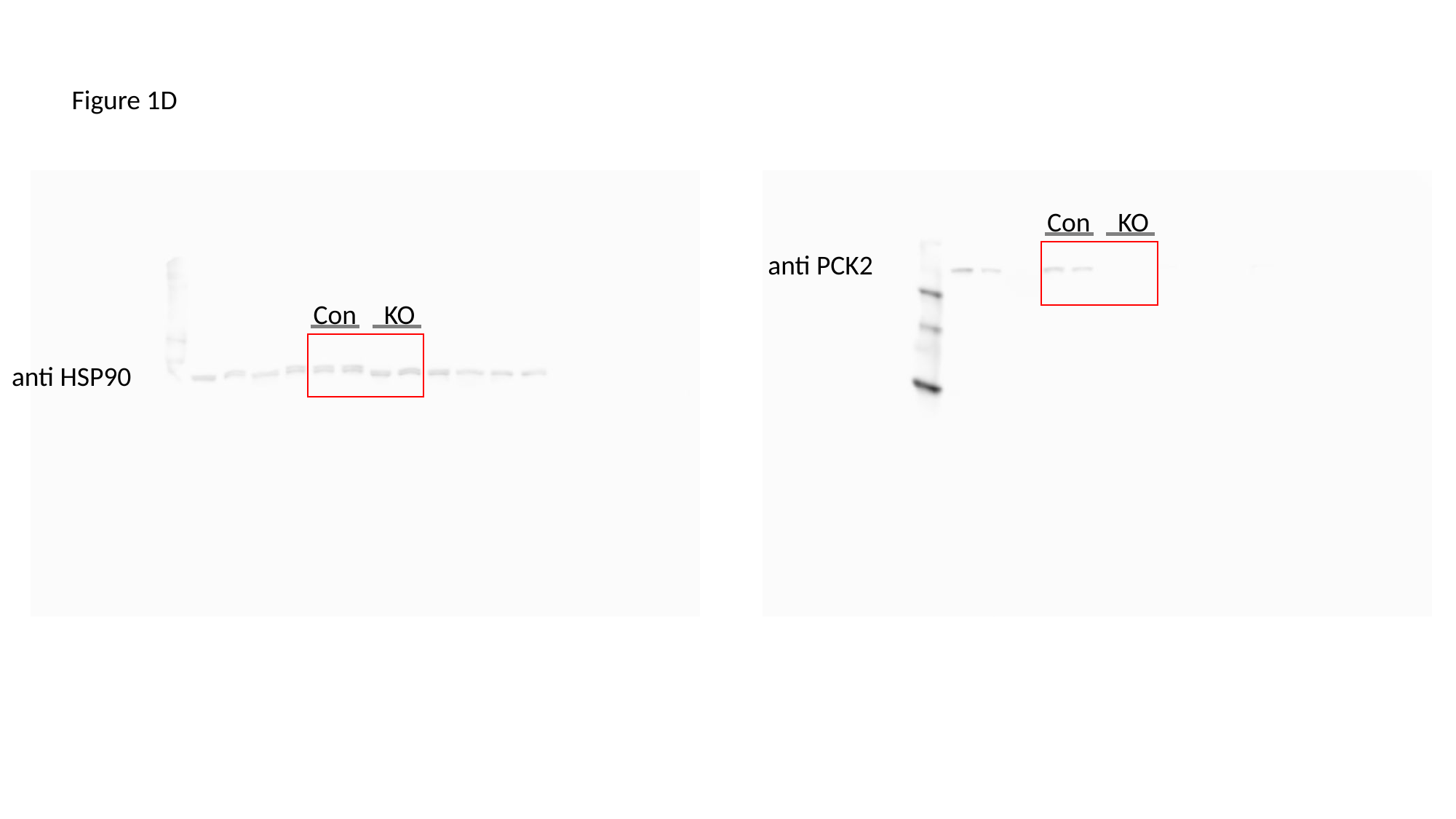

Figure 1D
KO
Con
anti PCK2
KO
Con
anti HSP90
