# Supplementary figures and images for: β-cell deletion of the PKm1 and PKm2 isoforms of pyruvate kinase in mice reveals their essential role as nutrient sensors for the KATP channel
Source: eLife. 2022 Aug 23;11:e79422. doi: 10.7554/eLife.79422 (PMC9444242; doi:10.7554/eLife.79422)

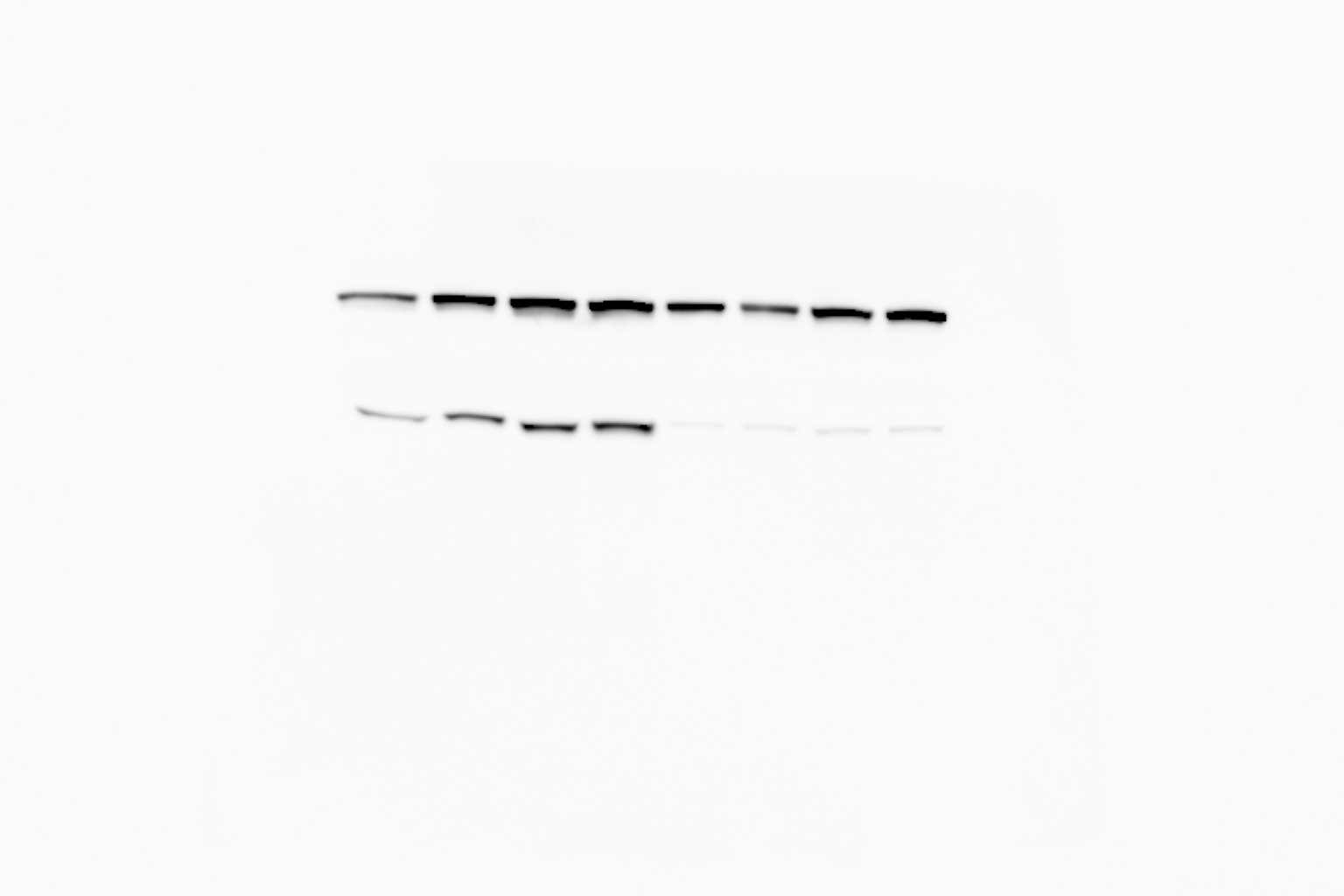

Supplement: Figure 1—source data 1. [file elife-79422-fig1-data1.zip › Figure 1-source data 1 western/Figure 1B 20190424_PKM1 KO wester anti PKM1_5.tif]

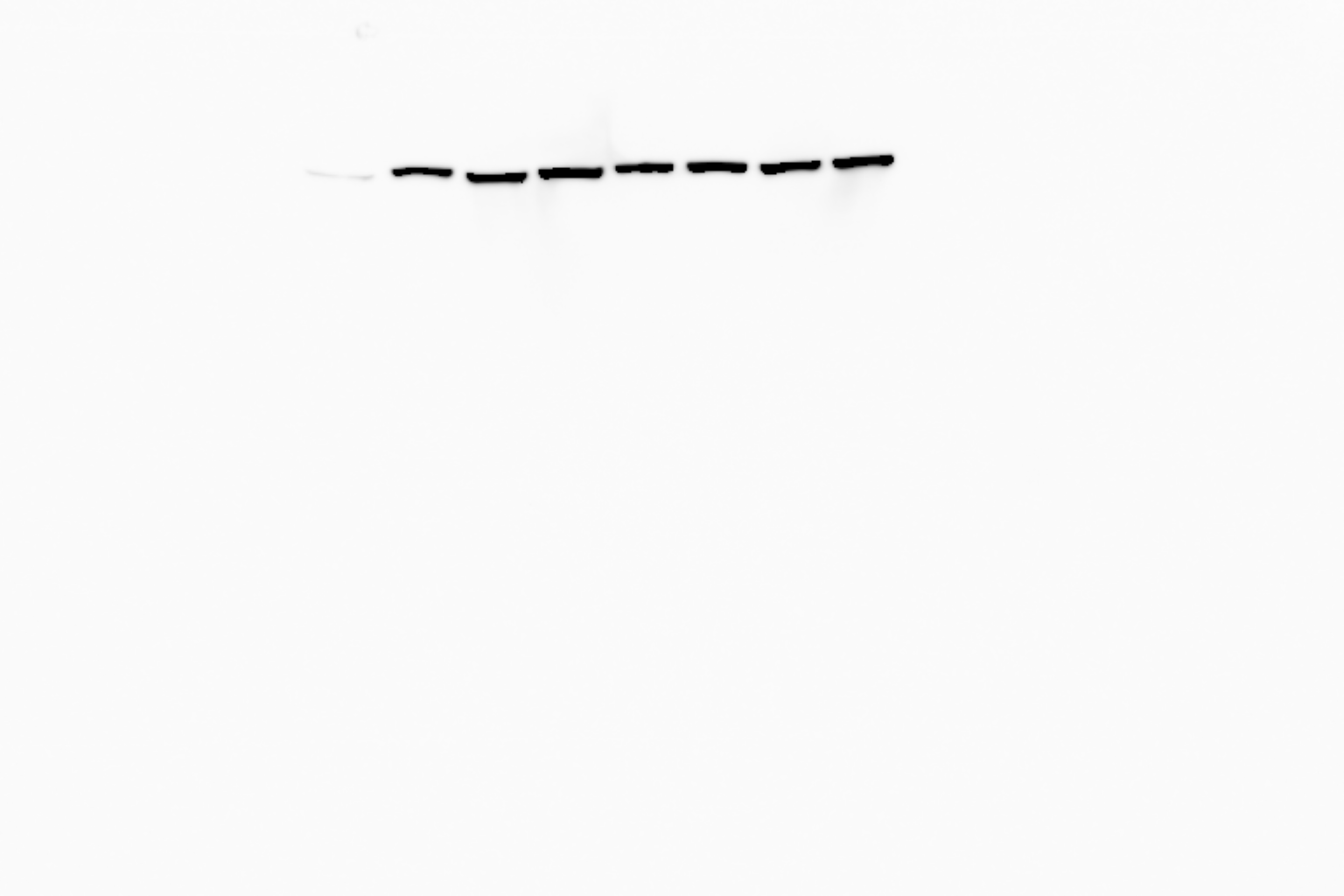

Supplement: Figure 1—source data 1. [file elife-79422-fig1-data1.zip › Figure 1-source data 1 western/Figure 1B 20190426_PKM1 KO western anti PKM2_9.tif]

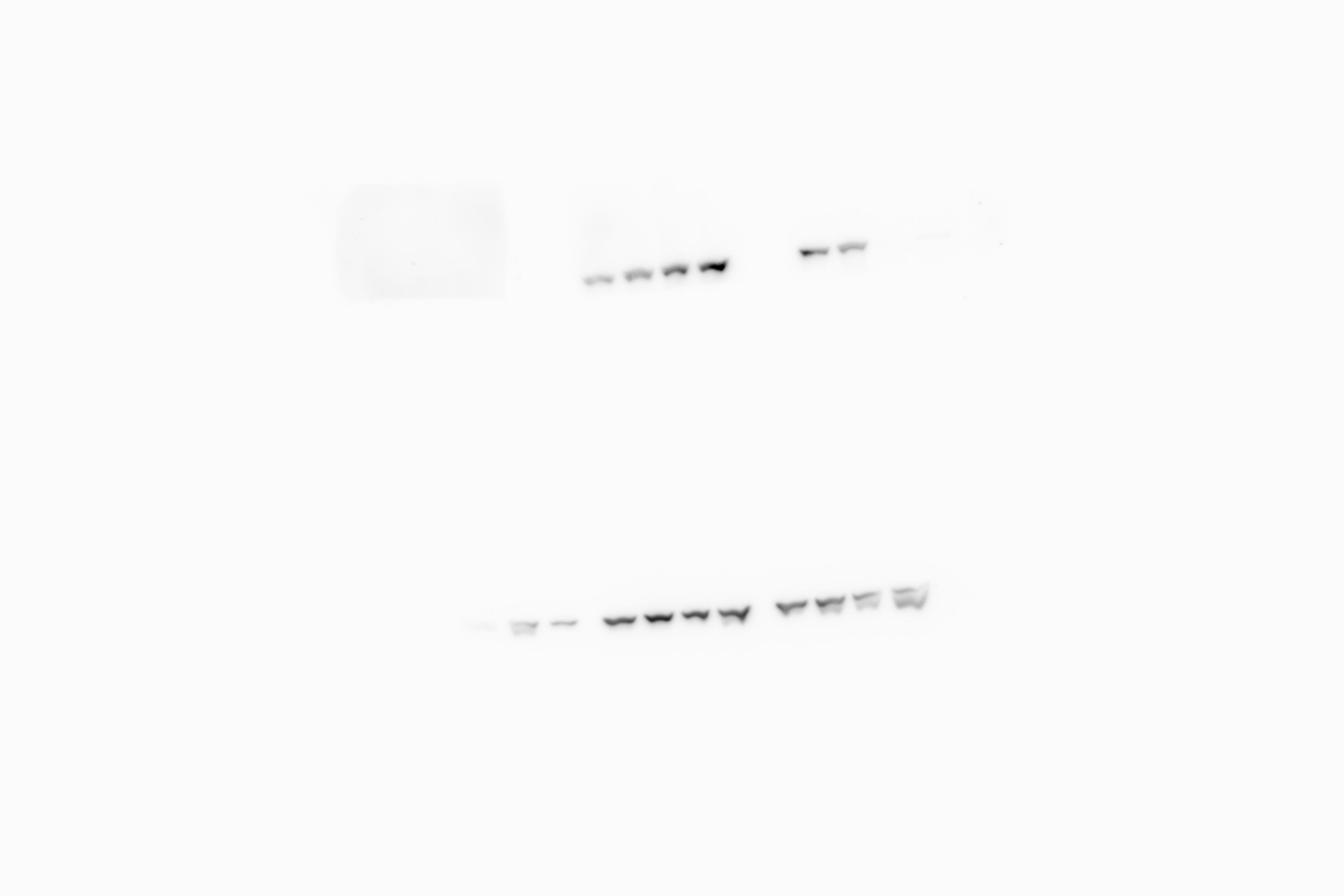

Supplement: Figure 1—source data 1. [file elife-79422-fig1-data1.zip › Figure 1-source data 1 western/Figure 1C 20160927_PKM2 KO 1119_12.tif]

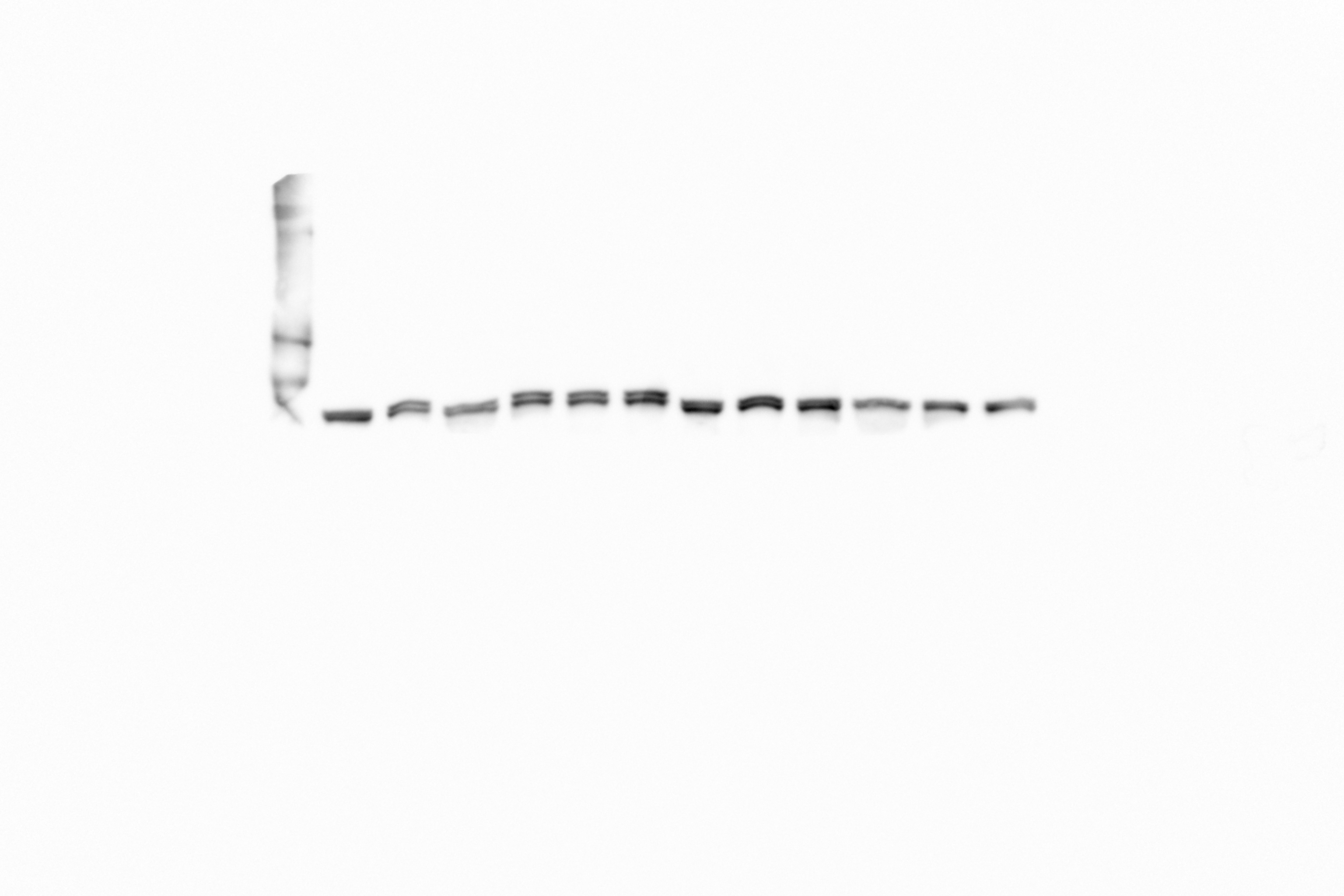

Supplement: Figure 1—source data 1. [file elife-79422-fig1-data1.zip › Figure 1-source data 1 western/Figure 1D 20210429_HSP90 for PCK2 blot_4_4.tif]

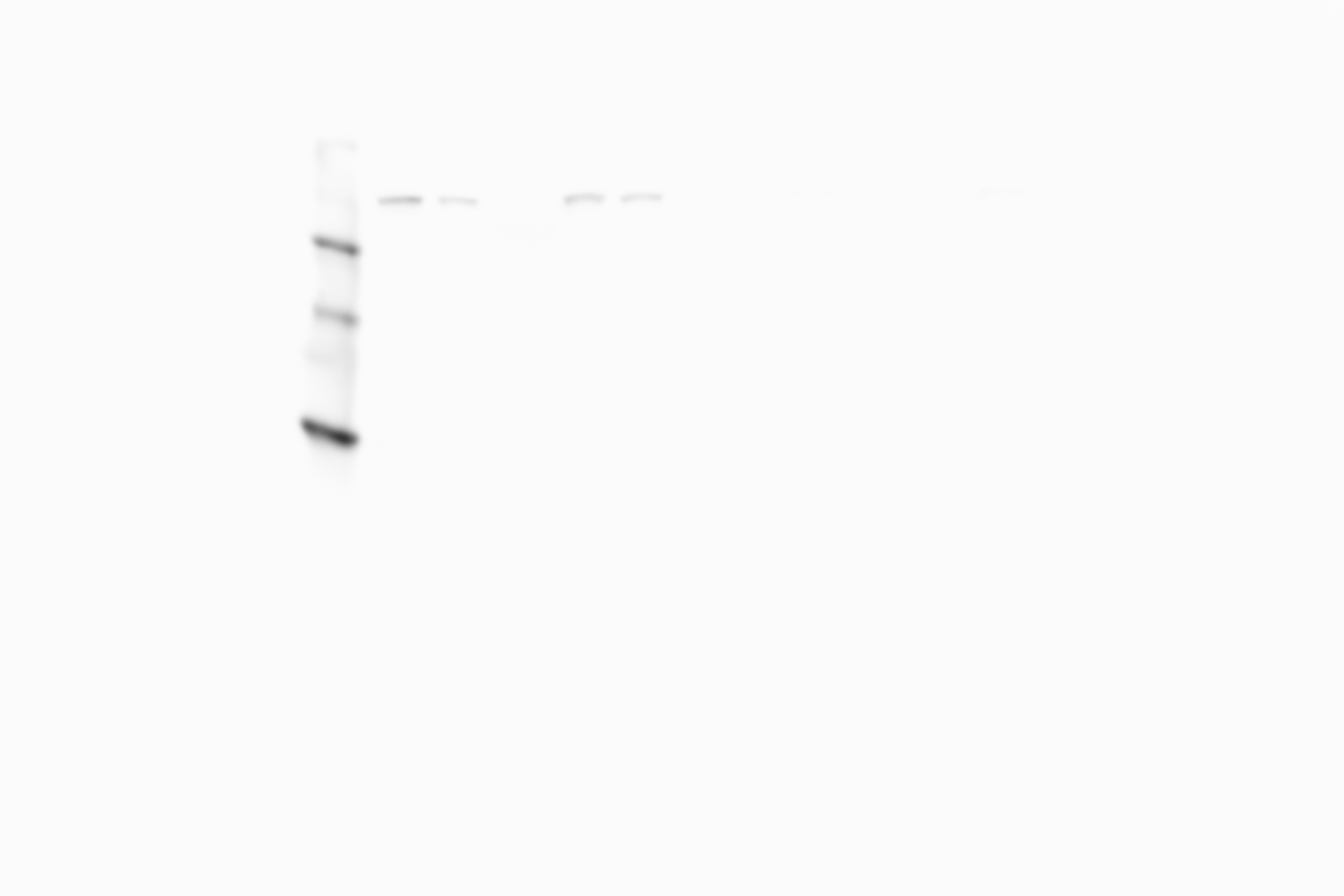

Supplement: Figure 1—source data 1. [file elife-79422-fig1-data1.zip › Figure 1-source data 1 western/Figure 1D 20210429_PCK2_14.tif]
